# Factors associated with positive attitude towards hypertension control in Hawassa city administration: Community based cross‐sectional study

**DOI:** 10.1002/hsr2.779

**Published:** 2022-08-14

**Authors:** Tsegab Paulose, Zerish Z. Nkosi, Misganu Endriyas

**Affiliations:** ^1^ Department of Health Studies University of South Africa Ethiopia Regional Learning Center Addis Ababa Ethiopia; ^2^ Department of Health Studies University of South Africa Pretoria South Africa; ^3^ Health Research and Technology Transfer Directorate SNNPR Health Bureau Hawassa Ethiopia

**Keywords:** attitude, hypertension, knowledge, prevention and control, risk factors

## Abstract

**Background and Aims:**

In low‐income countries where there is shortage of appropriate medical care to manage hypertension (HTN), understanding dynamics of communities' knowledge and attitude to prevent through lifestyle is crucial. Despite this fact, there was limited information on levels of awareness and attitude towards HTN and its prevention in the study setting. So, this study was conducted to assess level and factors associated with positive attitude in Hawassa city, Southern Ethiopia.

**Methods:**

A community level study using cross‐sectional design was done in Hawassa city administration in 2017. Six hundred and twelve respondents were selected using a multi‐stage sampling technique. Knowledge and attitude were assessed using multiple questions and responses were categorized considering mean as cutoff points. Descriptive statistics and binary logistic regression analysis at 95% confidence interval (CI) were performed.

**Results:**

The level of mean score of knowledge was 62.7% (SD = 22.2) [95% CI: 60.9–64.4] while that of attitude was 68.1% (SD = 8.8) [95% CI: 67.4–68.8]. Divorced/widowed respondents were 73% less likely to have positive attitude as compared to married respondents (adjusted odds ratio [AOR] 95% CI: 0.27 [0.14–0.51], *p*‐<0.001). Respondents who attended primary, secondary and higher education were 2.84 times (AOR 95% CI: [1.48–5.42], *p* 0.002), 5.59 times (AOR 95% CI: [2.87–10.89], *p*‐<0.001) and 9.28 times (AOR 95% CI: [4.39–19.65], *p*‐<0.001) more likely to have positive attitude as compared to those who cannot read and write. Moreover, respondents who have good knowledge were 2.24 times (AOR 95% CI: [1.49–3.37], *p*‐<0.001) more likely to have positive attitude as compared with those who have poor knowledge.

**Conclusion:**

The overall levels of knowledge and attitude related to HTN and its prevention were moderate but not adequate to bring lifestyle modifications required to prevent and control HTN. Health promotion activities should be strengthened to improve awareness and attitude that are pillars to bring lifestyle modification practices.

**Abbreviations**
DM: diabetes mellitus
HTN: hypertension

## 1 BACKGROUND

Globally, about 1.13 billion people have elevated blood pressure or have hypertension (HTN).^1^ The prevalence is highest (27%) in WHO African Region due mainly to an increase in HTN risk factors.^1^

Different studies^2^, ^3^, ^4^, ^5^, ^6^, ^7^ reported physical inactivity, low fruit intake, obesity, smoking, family history, age, salt intake, alcohol, sex, chewing kchat, educational status, fat consumption, diabetes, marital status, occupation residence, and/or income as risk factors of HTN. Increasing urbanization, unhealthy eating habits, and sedentary lifestyles are also contributing to the increment of the disease.^8^, ^9^

A STEPS survey conducted in 2015 reported that the prevalence of HTN in Ethiopia was 15.6% (95% confidence interval [CI]: 14.4%–16.9%).^10^ Systematic review and meta‐analysis of studies conducted on HTN in Ethiopia showed that pooled prevalence of HTN was 21.81% (95% CI: 19.20–24.42).^11^

For this alarming health condition especially in low‐income countries where there is shortage of appropriate medical care, understanding dynamics of communities' knowledge and attitude to prevent through lifestyle modification is crucial.^12^ Despite this fact, there was limited information on levels of awareness and attitude to behavioral change in the study setting. So, this study was conducted to assess knowledge and attitude towards HTN prevention and factors associated with positive attitude in Hawassa city, Southern Ethiopia.

## 2 METHODS

In 2017, community‐based cross‐sectional study was conducted in Hawassa City Administration, Sidama Region, Ethiopia. From 2005 census projection, the city had population of 351,567 in a total of eight sub‐cities from which 250,777 were living in urban area and 100,790 were living in peri‐urban area. The peri‐urban area refers a setting which is partly urban and partly rural.

Sample size was calculated using single population formula assuming proportion of people with a positive attitude 50% (to maximize sample size), 5% margin of error, and 80% power at 95% CI. Considering the design effect of 1.5 and after adding 10% nonresponse rate, the final sample size was 633.

To select the study participants, a multi‐stage sampling technique was used. First, from eight sub‐cities in the city administration, two urban and one peri‐urban were selected randomly. In the second stage, a total of nine *kebeles* (three *kebeles* from each sub‐city) were selected using simple random sampling. *Kebeles* are lowest administrative structure in the study setting. Considering population size, the calculated sample size was allocated proportionally to selected kebeles. In the third stage, households were selected randomly from lists of households in kebeles. And finally, one random adult resident from each household fulfilling inclusion criteria were included in the study. Age of 30 years or more and residing for 6 months or more in the city were considered as inclusion criteria.

Health Belief Model (HBM) from the individual‐level theories was used to assess behavior and attitude. The Health Belief Model contains concepts that predict why people will act to prevent conditions, what barriers hinder them, how do they feel like the seriousness of the condition and their susceptibility, and what promotes them to take action. The model is constructed of six blocks. These are the perceived severity/threat, perceived susceptibility, perceived benefit, perceived barriers, cues to action, and self‐efficacy.^13^

The questionnaire comprised socio‐demographic variables, 10 questions on knowledge about HTN and its prevention, and 37 questions to measure attitude. Attitude questions included eight questions of perceived susceptibility, 11 questions of perceived severity, five questions of perceived benefit of practicing HTN prevention activities, seven questions of perceived barriers to practice HTN prevention activities, five questions of cues to taking action to prevent HTN, and one question of self‐efficacy.

To ensure validity in the study, content and criterion approaches were used. Pre‐testing in a setting other than actual data collection and appropriate modifications were performed to ensure reliability.

Data were managed by using Statistical Package for the Social Sciences (SPSS) for Windows version 25. Descriptive statistics were performed to describe study participants. The wealth index was constructed by running principal component analysis to describe economic status of household. Both scores of knowledge and attitude were summed and means were used as cutoff point to categorize responses as good or poor knowledge and positive or negative attitude, respectively. Knowledge scores above the mean were considered good knowledge. Similarly, attitude scores above the mean were considered positive attitude. Predictors of positive attitude were assessed using binary logistic regression at 95% CI (two‐tailed). *p*‐value of 0.2 or less was considered at bivariate analysis level and *p*‐value of less than 0.05 was used in the final model.

After getting ethical approval from Research and Ethics Committee of University of South Africa (Ref: REC‐012714‐039), permission letter to conduct study was obtained from Southern Nations Nationalities and Peoples' Region health bureau and Hawassa City Administration health department. Written consent was obtained from study participants. Respondents needing medical care were linked to health facilities.

## 3 RESULTS

### 3.1 Sociodemographic characteristics

Six hundred and twelve respondents completed the investigation with response rate of 96.5%. About half, 327 (53.4%), of participants were male and about two‐thirds, 408 (66.7%), were from urban setting. Moreover, about one‐third, 199 (32.5%), were employed and majority, 500 (81.7%), were married (Table 1).

**Table 1:** Sociodemographic characteristics of respondents, Hawassa, 2017

Variable	Categories	Frequency	Percent
Residence	Urban	408	66.7
	Peri‐Urban	204	33.3
Sex	Male	327	53.4
	Female	285	46.6
Age	31–40	260	42.5
	41–50	152	24.8
	51–60	92	15.0
	61+	108	17.6
Marital status	Married	500	81.7
	Divorced/widowed	83	13.6
	Single	29	4.7
Educational status	Cannot read and write	139	22.7
	Read and write only	98	16.0
	Primary (1–8)	84	13.7
	Secondary (9–12)	108	17.6
	Diploma and above	183	29.9
Occupation	Employee	199	32.5
	Daily‐laborer	53	8.7
	Merchant	165	27.0
	Housewife	118	19.3
	Retired	59	9.6
	Others	18	2.9
Wealth index	Lowest	170	27.8
	Second	103	16.8
	Middle	117	19.1
	Fourth	161	26.3
	Highest	61	10.0
Reported family history of hypertension	No	528	86.3
	Yes	84	13.7
Reported presence of DM	No	561	91.7
	Yes	51	8.3

Abbreviation: DM, diabetes mellitus.

### 3.2 Knowledge about HTN and its prevention

A majority, 587 (95.9%), of participants know that HTN can cause sudden death. The lowest scores were recorded for questions on normal blood pressure, 257 (42.0%), and place where blood pressure can be measured, 248 (40.5%) (Table 2). From the total of ten questions, the mean score was 62.7% (SD = 22.2) (95% CI: 60.9–64.4), and 325 (53.1%) respondents scored above mean and categorized as good knowledge.

**Table 2:** Knowledge about HTN and its prevention, Hawassa, 2017

Knowledge questions	Frequency	Percent
Ever had heard about HTN	508	83.0
Ever received information on HTN from health worker	406	66.3
HTN cannot be transmitted from person to another	406	66.3
HTN can be prevented	405	66.2
Know at least two places where BP is measured	248	40.5
HTN is hereditary	388	63.4
HTN is a lifelong disease	348	56.9
HTN may cause sudden death	587	95.9
Know the normal blood pressure value	257	42.0
Know at least three risks factors of HTN (out of six listed)	484	79.1

Abbreviation: HTN, hypertension.

### 3.3 Attitude

From a total of 37 questions considered, 32 questions had five scale while five questions had four, making maximum score to be 180. The mean level of attitude score was 68.1% (SD = 8.8) [95%CI: 67.4–68.8], and 319 (52.1%) participants scored above mean and considered as positive attitude.

### 3.4 Factors associated with positive attitude

Multivariable binary logistic regression showed that marital status, educational status and overall knowledge about HTN and its prevention were associated with positive attitude at 95% CI (Table 3).

**Table 3:** Bivariate and multivariable binary logistic regression of positive attitude towards HTN prevention, Hawassa, 2017

Variables	Attitude		Crude odds ratio 95% CI	*p*‐value	AOR 95% CI*	*p* value
	Negative no (%)	Positive no (%)				
Residence						
Urban	194	214	1			
Peri‐urban	99	105	0.96 [0.69–1.35]	0.82		
Sex						
Male	146	181	1			
Female	147	138	0.76 [0.55–1.04]	0.08		
Age						
31–40	108	152	1			
41–50	74	78	0.75 [0.50–1.12]	0.16		
51–60	48	44	0.65 [0.40–1.05]	0.08		
≥ 61	63	45	0.51 [0.32–0.80]	0.003		
Marital status						
Married	224	276	1			
Divorced/widowed	60	23	0.31 [0.18–0.52]	<0.001	0.27 [0.14–0.51]	<0.001
Single	9	20	1.80 [0.80–4.04]	0.15	1.23 [0.47–3.19]	0.67
Educational status						
Cannot read and write	108	31	1			
Read and write only	71	27	1.33 [0.73–2.41]	0.36	1.08 [0.56–2.09]	0.80
Primary (1–8)	42	42	3.48 [1.94–6.25]	<0.001	2.84 [1.48–5.42]	0.002
Secondary (9–12)	35	73	7.37 [4.12–12.81]	<0.001	5.59 [2.87–10.89]	<0.001
Diploma and above	37	146	13.74 [8.03–23.55]	<0.001	9.28 [4.39–19.65]	<0.001
Occupation						
Employed	56	143				
Daily‐laborer	36	17	0.18 [0.09–0.36]	<0.001		
Merchant	69	96	0.55 [0.35–0.84]	0.01		
Housewife	84	34	0.16 [0.09–0.26]	<0.001		
Retired	39	20	0.20 [0.11–0.37]	<0.001		
Others	9	9	0.39 [0.15–1.04]	0.06		
Wealth Index						
Lowest	111	59	1			
Second	62	41	1.24 [0.75–2.06]	0.40		
Middle	49	68	2.61 [1.61–4.24]	<0.001		
Fourth	55	106	3.63 [2.30–5.71]	<0.001		
Highest	16	45	5.29 [2.76–10.16]	<0.001		
Reported family history of hypertension						
No	255	273	1			
Yes	38	46	1.13 [0.71–1.79]	0.60		
Reported presence of DM						
No	276	285	1			
Yes	17	34	1.94 [1.06–3.55]	0.03		
Knowledge about HTN						
Poor	187	100	1			
Good	106	219	3.86 [2.76–5.40]	<0.001	2.24 [1.49–3.37]	<0.001

Abbreviation: CI, confidence interval; DM, diabetes mellitus; HTN, hypertension.

* Blank cells had no significant associations and left to minimize texts in table.

Divorced or widowed respondents were 73% less likely to have positive attitude as compared to married respondents (AOR 95% CI: 0.27 [0.14–0.51], *p* < 0.001). Respondents who attended primary, secondary and higher education were 2.84 times (AOR 95% CI: [1.48–5.42], p‐0.002), 5.59 times (AOR 95% CI: [2.87–10.89], *p* < 0.001) and 9.28 times (AOR 95% CI: [4.39–19.65], *p* < 0.001) more likely to have positive attitude as compared to those who cannot read and write. Moreover, respondents who have good knowledge were 2.24 times (AOR 95% CI: [1.49–3.37], *p* < 0.001) more likely to have positive attitude as compared to those who have poor knowledge about HTN and its prevention.

## 4 DISCUSSION

This study was done to assess knowledge about and attitude towards HTN and its prevention, and factors associated with positive attitude in Hawassa city. Key findings included that the level of mean score of knowledge was 62.7% (SD = 22.2) while that of attitude was 68.1% (SD = 8.8). Marital status, educational status and overall knowledge about HTN and its prevention were associated with having positive attitude towards HTN and its prevention at 95%CI.

Despite less attention that had been given to HTN in low‐income countries due to high burden of infectious diseases, HTN is now a prevalent health problem. One of our article on prevalence of HTN in study setting showed that it was 21.2% (95% CI: 18.1–24.7)^14^ and was comparable with results from systematic review and meta‐analysis on HTN in Ethiopia that indicated pooled prevalence of HTN to be 21.81% (95% CI: 19.20–24.42).^11^ The current paper adds communities' knowledge and attitude towards HTN and its prevention, which are base for lifestyle modifications.

The general assumptions of focusing on people's knowledge and attitude toward disease prevention and control is that if individuals consider themselves as susceptible to a condition capable of bringing serious consequences, they are likely to take action that they believe will reduce their risks.^12^ As HTN is increasing in low‐income countries like Ethiopia,^11^ where there is shortage of appropriate medical care to manage the disease at advanced stage, focusing on available prevention and control interventions is important. For this purpose, understanding dynamics of communities' knowledge about risk factors and attitude to prevent through lifestyle modifications is crucial.^12^

The overall level of knowledge about HTN (62.7%) in study setting is fair. Although almost all (95.9%) of respondents were aware of HTN that it can cause sudden death, gaps in knowledge that HTN is lifelong (56.9%), places where BP is measured (40.5%) and the normal blood pressure value (42.0%) were noted. Moreover, as much as 33.7% of respondents believe that HTN is transmitted from person to person while 33.8% think HTN cannot be prevented. Since these knowledge components are critical for the disease prevention, health education programs should be strengthened to address such knowledge gaps.

A community‐based follow‐up study indicated that health beliefs and attitudes have a significant association with lifestyle and an impact on lifestyle change. Respondents who underrate the risks and have a resistant attitude toward health promotion have unhealthier lifestyle including higher levels of cardiovascular risk factors as compared to others.^15^ In this study, the level of agreement to sets of attitude statements was 68.1%. The positive attitude (above mean score) was associated with marital status, educational status and overall knowledge about HTN and its prevention at 95% CI.

Evidence suggests that greater level of awareness positively impacts attitude toward disease prevention.^16^, ^17^ In this study, both educational status and overall knowledge about HTN and its prevention were positively associated with positive attitude. As educational status increased, the odds of having positive attitude consistently increased, except those who only read and write as compared to people who cannot read and write, probably indicating both population are similar as both groups did not attend formal education. As compared to respondents who cannot read and write, respondents who attended primary, secondary and tertiary level of education were 2.84 times, 5.59 times and 9.28 times more likely to have positive attitudes respectively. In addition, those who have good knowledge about HTN were 2.24 times more likely to have positive attitudes as compared to those who have poor knowledge.

Regarding marital status, respondents who divorced or widowed were 73% less likely to have positive attitude as compared to married respondents. Widowhood and divorce are significantly stressful events and are associated with psychological consequences.^18^ So, this negative attitude among divorced or widowed groups could be due to psychological impacts or less chance of sharing information with partner. A study from the same country, Ethiopia, also reported that divorced or widowed groups have less odds of positive attitude towards disease control as compared to married groups.^19^

In low‐income countries, the awareness about HTN, its management, and control remain very low^20^ and high amount of people are not diagnosed and unaware of the existing disease.^8^, ^21^, ^22^ Although lifestyle modifications are cost‐effective interventions to prevent HTN, there is poor compliance with lifestyle modifications even among hypertensive people as reported by a study done in southern Ethiopia.^23^ To improve lifestyle change practices, we recommend the health system to strengthen awareness creation and bringing positive attitude.

## 5 LIMITATIONS

Although we assessed knowledge and attitude related to HTN in the city considering urban and peri‐urban settings in sampling, we included permanent residents who lived in the area for at least 6 months and age greater than 30 years old. Another limitation of the study is that we did not measure lifestyle modifications.

## 6 CONCLUSION

The overall levels of knowledge and attitude related to HTN and its prevention and control were moderate but not adequate to bring lifestyle modifications required to prevent and control HTN. Marital status, educational status, and overall knowledge about HTN and its prevention were associated with having positive attitude towards HTN and its control. Health promotion activities should be strengthened to improve awareness and attitude that are pillars to bring lifestyle modification practices.

## AUTHOR CONTRIBUTIONS

**Tsegab Paulose**: Conceptualization; data curation; formal analysis; funding acquisition; investigation; methodology; supervision; validation; writing—original draft; writing—review & editing. **Zerish Zethu Nkosi**: Conceptualization; methodology; validation; writing—review & editing. **Misganu Endriyas**: Formal analysis; methodology; writing—original draft; writing—review & editing.

## CONFLICTS OF INTEREST

The authors declare no conflicts of interest. All authors declare that this study is original. All authors have read and approved the final version of the manuscript. TP had full access to all of the data in this study and takes complete responsibility for the integrity of the data and the accuracy of the data analysis. TP affirms that this manuscript is an honest, accurate, and transparent account of the study being reported; that no important aspects of the study have been omitted.

## TRANSPARENCY STATEMENT

The lead author (Tsegab Paulose) affirms that this manuscript is an honest, accurate, and transparent account of the study being reported; that no important aspects of the study have been omitted; and that any discrepancies from the study as planned (and, if relevant, registered) have been explained.

## Data Availability

The data that support the findings of this study are available from the corresponding author upon reasonable request.
